# Prospective Trial of Monocyte Count as a Biomarker of Hand-Foot Syndrome Among Patients With Soft Tissue Sarcomas Treated With Pegylated Liposomal Doxorubicin and Ifosfamide

**DOI:** 10.7759/cureus.24498

**Published:** 2022-04-26

**Authors:** Keith M Skubitz, Bruce R Lindgren, Evidio Domingo-Musibay, Edward Y Cheng

**Affiliations:** 1 Medicine, University of Minnesota, Minneapolis, USA; 2 Masonic Cancer Center, University of Minnesota, Minneapolis, USA; 3 Orthopaedics, University of Minnesota, Minneapolis, USA

**Keywords:** toxicity, liposome, pharmacokinetics, monocyte, hand-foot syndrome, drug-related side effect, sarcoma, doxorubicin, pegylated-liposomal doxorubicin

## Abstract

Pegylated liposomal doxorubicin (PLD) is widely used and can be used for prolonged periods, with the limiting toxicity usually being hand-foot syndrome (HFS). The pharmacokinetics of PLD is variable between patients, leading to variability in the risk of developing HFS. Dosing based on body surface area does not decrease variability in PLD clearance; thus, other predictive markers could be useful. The peripheral blood absolute monocyte count (AMC) has been suggested as a possible marker of both reticuloendothelial system function and PLD pharmacokinetics. The present study examined the AMC as a potential predictive biomarker in a prospective trial of pre-operative PLD combined with ifosfamide in soft tissue sarcomas (STSs). While our results suggest a relationship between pre-treatment AMC and PLD-induced HFS, the association did not reach statistical significance. The clinical utility of the AMC as a predictor of PLD-induced HFS appears limited, at least when given with ifosfamide.

## Introduction

The incorporation of drugs into liposomes is a useful approach to modifying the toxicity and efficacy of drugs. Doxorubicin is a widely used anti-cancer drug effective in many types of cancers, including sarcoma, breast cancer, and lymphoma but has important limiting toxicities, including cardiotoxicity and myelosuppression [1]. The incorporation of doxorubicin into pegylated liposomes significantly modifies these toxicities. The liposomes of pegylated liposomal doxorubicin (PLD) are coated with methoxypoly(ethylene glycol), which decreases uptake in the reticuloendothelial system (RES), resulting in a longer half-life in blood than non-pegylated liposomes [2-4]. It also limits distribution to certain body compartments, such as the myocardium [5]. The change in pharmacokinetics results in important changes to the toxicity profile of doxorubicin, including a marked reduction of cardiotoxicity and myelosuppression, as well as markedly decreased alopecia [6]. In addition, no pre-medication or growth factors are necessary, and anti-emetics are rarely needed. Studies have also demonstrated increased drug delivery to the tumor with PLD compared with free doxorubicin, presumably due to the increased vascular permeability of the neovasculature of the tumor [4, 7, 8]. In some cases, PLD has been more effective than free doxorubicin [9]. 

The main toxicities of PLD include mucositis and skin toxicity, commonly referred to as hand-foot syndrome (HFS), a low risk of an infusion reaction, and some fatigue. The infusion reaction is typically reflected by shortness of breath or low back pain during the first few minutes of PLD infusion during the first treatment. It has been suggested that these symptoms, which are associated with transient neutropenia, may reflect neutrophil sludging in the microvasculature as observed with hemodialysis neutropenia [10]. However, HFS is the main dose-limiting toxicity of PLD, and the risk of HFS at commonly used doses is not easily predicted. 

There is significant interpatient variability in PLD pharmacokinetics; in one study, there is up to 15-fold variability [11]. A longer half-life has been correlated with a higher risk of HFS [12]. Despite the historical use of body surface area (BSA) to dose chemotherapeutic agents, its ability to reduce intrapatient variability, including drug effect, is limited for most chemotherapeutic agents [13-16]. For PLD, one study found that BSA dosing did not decrease the large interpatient variability in PLD clearance [11]. In addition, PLD clearance decreases from cycles 1 to 3 [11]. Thus, identifying factors predictive of decreased clearance could be clinically useful.

Most nanoparticles are felt to be cleared by the RES, also known as the mononuclear phagocyte system. The circulating monocyte count has been suggested as a possible marker of RES function [17]. A positive association was found between blood monocyte phagocytosis, reactive oxygen species production, and PLD clearance in humans and several other species [17]. Monocyte quantity has been reported to affect the clearance of other liposomal drugs, and a reduction of peripheral blood monocyte count has been correlated with PLD clearance [11]. PLD itself can inhibit the RES and prolong PLD clearance, resulting in more drug exposure, an effect not seen with free doxorubicin or empty pegylated liposomes [18]. A study of 35 patients over age 70 also found high interpatient variability in PLD clearance (>10-fold range) and a correlation between monocyte count and PLD clearance, but only after three cycles. They also reported that PLD clearance is correlated with age, and a longer PLD half-life is correlated with the development of HFS [19]. Another retrospective study of 88 patients found that HFS is more common with subsequent cycles of PLD and older age [20].

The current study examined the relationship between absolute monocyte count (AMC) and the need for PLD dose reduction due to HFS in 63 patients in a prospective clinical trial of preoperative PLD and ifosfamide in soft tissue sarcoma (STS).

## Materials and methods

This study examined 63 evaluable patients with STS, receiving up to four cycles of preoperative chemotherapy with PLD and ifosfamide in a prospective clinical trial (ClinicalTrials.gov Identifier: NCT00346125, first registration 29/06/2006) [21]. The trial prospectively collected toxicity, drug dosing, and laboratory values, although the goal to examine the relationship between toxicity and blood cell counts was added later. In this regard, the current study is partly retrospective. The relationship between the AMC or the absolute neutrophil count (ANC) and dose reduction of PLD due to HFS was examined. The dose-limiting toxicity of PLD is HFS. The definition of a need for a dose reduction was whether the treating clinician, who was the same for all patients, deemed it necessary at the time due to HFS. The University of Minnesota IRB approved the study. Informed consent was obtained from all subjects, and all methods were carried out in accordance with relevant guidelines and regulations. Patients were treated from 2006 to 2014 and were >18 years old with high-grade (FNCLCC grade 3) STS of the extremities or body wall whose tumors were greater than 5 cm in maximum diameter. Patients received preoperative chemotherapy followed by wide surgical excision of their tumor and subsequent external beam radiation; the goal of the clinical trial was to correlate treatment response with early positron emission tomography (PET) changes. The current study represents a planned secondary endpoint. The chemotherapy regimen was PLD at 45 mg/m2 IV on day 1 every 28 days, with ifosfamide given by continuous intravenous infusion (CIVI) at 1.5 g/m2/day for six days (total dose over six days of 9 g/m2), in conjunction with mesna 1.5 g/m2/day for seven days [21, 22]. Granulocyte colony-stimulating factor (G-CSF) was used prophylactically. 

Statistical methods 

The pre-treatment AMC and ANC values before the first chemotherapy dose were summarized by the mean, SD, minimum and maximum, separately by those who did and did not require a PLD dose reduction after the first dose or after any of the four planned doses. The two-sample, two-tailed t-test compared the baseline AMC and ANC levels between these two groups defined by whether or not the dose was reduced during the 4-dose regimen. The results were reported as the mean difference between groups and the 95% CI. Two-sided p-values less than 0.05 were considered statistically significant. All statistical analysis was performed using SAS version 9.4 (SAS Institute Inc., Cary, NC).

## Results

Sixty-five patients were enrolled in the study, two of whom withdrew due to early progression and were excluded from the analysis. After the first cycle of PLD and ifosfamide, 8/63 patients developed sufficient HFS to require PLD dose reduction. After all four planned cycles of neoadjuvant therapy, 19/63 patients developed sufficient HFS to require PLD dose reduction. The AMC decreased following treatment with PLD and ifosfamide. Based on the two-sample t-test, there was a trend for the AMC to be lower when a subsequent dose reduction of PLD was required, but this did not reach conventional statistical significance (p <0.05; Table 1). Due to the skewed distribution of AMC values, the analysis was also performed using a logarithmic scale, but the results of the t-test were very similar (not shown). Similarly, ANC had no demonstrable effect on the need for dose reduction (Table 1).

**Table 1:** Pre-chemotherapy AMC and ANC levels as predictors of dose reduction for subsequent therapy. AMC: Absolute monocyte count; ANC: Absolute neutrophil count.

	Mean (SD)	Min/Max	Mean Group Difference	95% CI of Mean	P-value*
AMC			184.0	-39.5, 407.5	0.105
No reduction after 1^st^ cycle (n = 57)	696.5 (305.9)	300/1900			
Reduction after 1^st^ cycle (n = 8)	512.5 (203.1)	100/700			
ANC			446.5	-1359.8, 2252.8	0.623
No reduction after 1^st^ cycle (n = 57)	5596.5 (2479.7)	2200/13900			
Reduction after 1^st^ cycle (n = 8)	5150.0 (1548.3)	3100/7600			
AMC + ANC			630.5	-1328.8, 2589.8	0.523
No reduction after 1^st^ cycle (n = 57)	6293.0 (2685.5)	2700/15700			
Reduction after 1^st^ cycle (n = 8)	5662.5 (1731.2)	3200/8300			
AMC			104.4	-19.0, 227.9	0.096
No reduction after 4 cycles (n = 44)	688.6 (293.5)	300/1900			
Reduction after 4 cycles (n = 19)	584.2 (186.4)	100/900			
ANC			933.9	-241.4, 2109.1	0.117
No reduction after 4 cycles (n = 44)	5702.3 (2346.0)	2200/12000			
Reduction after 4 cycles (n = 19)	4768.4 (1544.9)	2800/8200			
AMC + ANC			1038.3	-222.0, 2298.5	0.105
No reduction after 4 cycles (n = 44)	6390.9 (2512.9)	2700/13100			
Reduction after 4 cycles (n = 19)	5352.6 (1666.5)	3200/9100			

## Discussion

PLD is used widely, and the main dose-limiting toxicity is HFS. Studies have suggested that AMC may be useful in predicting PLD pharmacokinetics and HFS. In this prospective study of pre-operative PLD and ifosfamide, we found a trend toward the need for dose reduction of PLD after the first cycle and a lower AMC and a dose reduction after any cycle and a lower AMC. However, this did not reach statistical significance (p = 0.105 and 0.096, respectively). As neutrophils also interact with PLD, both in vivo and in vitro [10], we also tested for a relationship between ANC and the need for dose reduction due to HFS. While there was a trend toward a need for dose reduction after the first cycle or after any cycle and a lower ANC, statistical significance was again not reached (p = 0.623 and 0.117, respectively). One limitation of the study is that PLD was given with ifosfamide, and the addition of the second drug could have masked any effect of the AMC on PLD toxicity by causing myelosuppression, which is not common toxicity of PLD.

In this prospective study, PLD was given with ifosfamide, which may have altered the relationship between AMC and HFS. We did not measure pharmacokinetics, but the outcome was the clinically relevant measure of the need for dose reduction due to HFS (as determined by the PI). Our data suggest a relationship between pre-treatment AMC and PLD-induced HFS that could reflect pharmacokinetics. However, this effect is unlikely to have practical clinical significance, at least when PLD is combined with ifosfamide.

In addition to dose reduction, another method that may help decrease oral and skin toxicity of PLD is cooling the mouth or cutaneous sites during and after administration, as PLD distribution to tissues is largely dependent on blood flow and vascular permeability of the tissue (about half of the administered PLD leaves the blood in the first ~50 hours). Additionally, some oral glutamine preparations have been shown to decrease chemotherapy-induced mucositis [23-26].

## Conclusions

HFS is the main dose-limiting toxicity of PLD, and the risk of HFS in practice is not easily predicted. Most liposomes are felt to be cleared by the mononuclear phagocyte system, also known as the RES. The AMC has been suggested as a possible marker of RES function and has been reported to correlate with the clearance of other liposomal drugs, including PLD clearance. This study examined the relationship between AMC and the need for PLD dose reduction due to HFS in patients receiving preoperative PLD and ifosfamide in STS. Our data suggest a relationship between pre-treatment AMC and PLD-induced HFS that could reflect pharmacokinetics. However, this effect is unlikely to have practical clinical significance, at least when PLD is combined with ifosfamide. The concomitant use of ifosfamide with PLD in this prospective study may have altered the relationship between AMC and HFS by causing myelosuppression, not typically seen with PLD alone.
